# Improved neonatal survival through economically sustainable reorganization of a neonatal care unit in a developing country: 7-year experience in the *Centre Medical Saint Camille* (CMSC) of Ouagadougou, Burkina Faso

**DOI:** 10.1007/s12682-012-0133-y

**Published:** 2012-12-19

**Authors:** Paolo Ernesto Villani, Alessandra Ricchini, Agnes Thombiano, Paul Ouedraogo, Donatella Cattarelli, Maria Paola Chiesi, Salvatore Pignatelli, Virginio Pietra, Autino Beatrice, Giovanna Mescoli, Richard Fabian Schumacher

**Affiliations:** 1 Dipartimento Materno-Infantile, UO TIN, Neonatologia e Nido, AO “C.Poma”, Mantua, Italy; 2 Dipartimento Materno-Infantile, UO TIN, AO Spedali Civili Brescia, Brescia, Italy; 3 Dipartimento Materno-Infantile, UO Pediatria, AO di Desenzano del Garda (BS), Brescia, Italy; 4 Patologia Neonatale “G. Moscati”, CMSC Ouagadougou, Ouagadougou, Burkina Faso; 5 Fondazione Chiesi, Parma, Italy; 6 Medicus Mundi Italia, Brescia, Italy; 7 Dipartimento Materno-Infantile, Clinica Pediatrica, AO Spedali Civili, Università di Brescia, Brescia, Italy

## Background

Every year approximately 3.7 million neonatal deaths occur worldwide plus an additional 3.3 million stillbirths. Thirty-eight percent of all under 5-year-old deaths are concentrated in the first 28 days of life, 75 % of them in the first 7 days, making the first week the most dangerous period of a lifetime. Sub-Saharan Africa remains the most dangerous region to be born, here 1.16 million babies do not survive more than 28 days and every year, half a million babies die within the first 24 h of life [1]. Just three causes account for 86 % of those deaths: asphyxia, pre-maturity, and severe infections like tetanus, pneumonia, and diarrhea. In fact, it is estimated that serious infections represent 36 % of all neonatal deaths [2], making hygiene a priority. We here report our experience of 7 years of collaboration with the Neonatal Care Unit of the CMSC of Ouagadougou, Burkina Faso, one of the world’s poorest countries.

Over 70 % of the 15.4 million inhabitants of Burkina Faso live below the poverty line. Schooling is sparse and health facilities are scarce [3]. Many people, especially children, are severely malnourished and do not have access to clean water, secure food supply, or medical care [4]. Many of these children die from treatable or even preventable infectious diseases such as diarrhea, pneumonia, malaria, HIV, TB, measles, and tetanus [5].

**Fig. 1:**
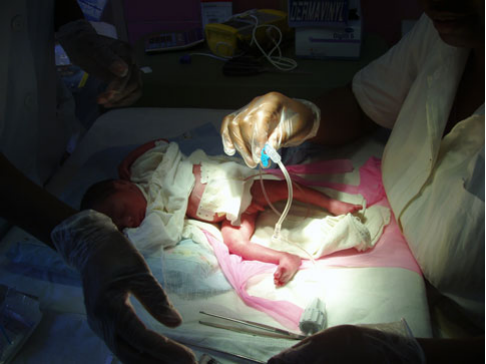
Neonatal pathology “Saint Joseph Moscati”, Centre Medical Saint Camille, Ouagadougou, 2010: umbilical vein catheterization procedures preparation by nurses

**Fig. 2:**
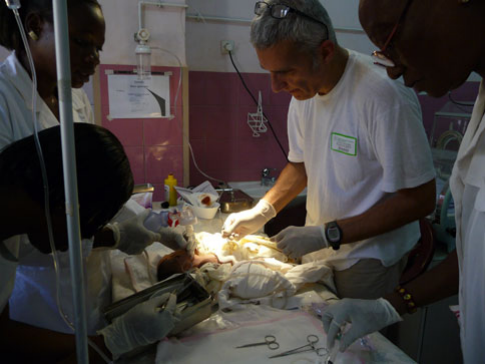
Neonatal pathology “Saint Joseph Moscati”, Centre Medical Saint Camille, Ouagadougou, 2010: umbilical vein catheterization in a intubates and ventilates with bag and mask neonate a term

**Fig. 3:**
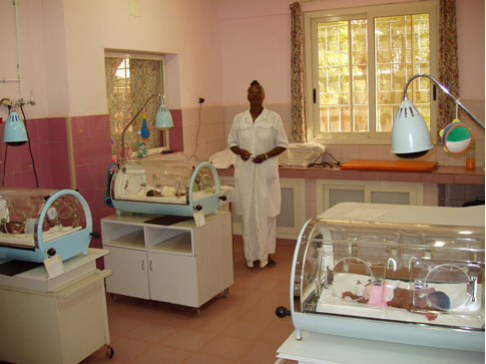
Neonatal pathology “Saint Joseph Moscati”, Centre Medical Saint Camille, Ouagadougou, 2011: the head nurse, Miss Thombiano Agnes

**Fig. 4:**
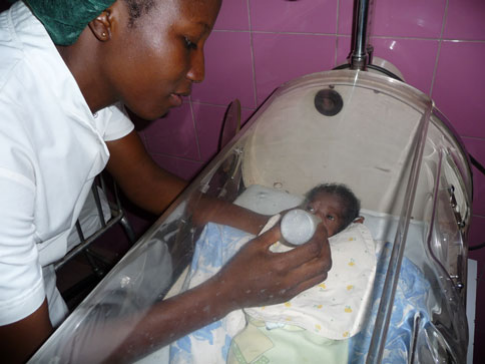
Neonatal pathology “Saint Joseph Moscati”, Centre Medical Saint Camille, Ouagadougou, 2009: enteral nutrition in premature

In 2004, the Center St. Camille comprised a maternity ward where more than 5,000 babies were delivered every year and a Neonatal Care Unit equipped with 12 incubators sent from Italy during the early 1980s. 1,200 babies were admitted each year to these incubators and to an additional 50 neonatal cots (all in one room). Oxygen was available from two 20 L pressure tanks. A small room for invasive procedures was available, and the center had a day-time laboratory and a small X-ray facility. Cardiologists equipped with sonography and fetal sonography were available twice weekly (Figs. 1, 2, 3, 4).

Country-wide, the CMSC is the only unit specializing in the care of premature newborns and is known beyond the borders of Burkina Faso. In fact, patients come also from neighboring countries such as Mali, Mauritania, and Sierra Leone.

Since 2005, Medicus Mundi Italy, a section of Medicus Mundi International, a Non-Governmental Organization (NGO), officially recognized by the WHO, as a specialized health organization, has an ongoing collaboration with the CMSC. With the aim to improve survival of the Neonatologic Unit, Neonatologists, and specialized nurses from the University of Brescia, “Spedali Civili” Hospital of Brescia and “Carlo Poma” Hospital in Mantova in Italy volunteered to be involved in short but frequent regular mission working closely with local staff. The main objective of the intervention was to train local nurses, working closely to them, teaching them how to monitor, maintain assistance, and ensure appropriate treatments to babies. To this end, it is essential to adapt modern neonatal care protocols for premature or otherwise ill babies to the highest achievable standard, in this extremely poor setting which is characterized by very limited technological resources.

The aim of the present contribution is to report on time trends of survival, covering the period in which the intervention based on improving levels of neonatal cares was carried out.

## Methods

### The setting

Given the lack of equipment, the first approach was to train and educate the available local personnel, consisting of the head nurse who was trained 20 years ago in Italy, 5 professional nurses, 16 auxiliary nurses, and 1 cleaning woman.

There is no dedicated physician for the unit, but during the day, a pediatrician in training is in the nearby pediatric unit. We were able to establish a stable oxygen supply by providing an oxygen concentrator, thus avoiding running out of oxygen which occurred frequently during nights and on week ends. Oxygen saturation measurements were introduced.

### Mode of action

During 12 missions (9 physicians and 3 nurses) made so far, emphasis was put on:

- correct evaluation of anthropometric parameters (weight, lengths, and head circumference), vital signs (heart rate, blood pressure, respiratory rate, and oxygen saturation), and clinical parameters such as fluid intake, urine output, and bowel movements. To record these parameters a medical chart was designed and a procedure to guarantee continuity of care during shift change of nurses was established.
- Each patient was assigned to one specified nurse, thus allowing for clear responsibility and accountability. Improved hygiene procedures were introduced ensuring the cleanliness of environments and the measures on newborns and operators [6]. In particular the washing of the hands before every activity, the disinfection of the skin of the babies, the use of the sterile gloves in the more invasive procedures such as catheterization of the umbilical vessels of peripheral veins. All procedures are recorded on appropriate signs posted in the wards.
- Development of easy-to-use procedures like targeted feeding, fluid intake, oxygen administration, and therapeutic protocols for the most common pathologies: strategies for antibiotic therapy, treatment of apnea, and seizures.
- Training courses focused on the care of the premature baby starting with delivery room resuscitation [7] and correct use of monitors to assess vital signs, but also a detailed explanation of the postures best to improve spontaneous breathing and the importance of adequate nutrition.
- The head nurse of the Neonatal Unit was invited for a stage at the Neonatal Intensive Care Unit of Brescia.
- Periodic reports by e-mail and telephone conferences to discuss clinical cases.

Improvement projects were already in place and continued to encourage immediate breast-feeding after birth by staff in the delivery room and through home visits of the mothers during the first week after delivery of healthy newborns.

The same staff also runs the outpatient clinics for all babies born at CMSC, including those discharged from the Neonatal Care Unit, where 20–40 patients are seen every day. The Neonatal Care Unit was also integrated in a national pilot project for the Prevention of Mother to Child Transmission of HIV that had started in 2003.

Furthermore during the intervention four additional nurses were hired and a local physician started his specialty training in pediatrics at the University Children’s Hospital in Brescia. He is now in his last year of residency and is planning to move back to Ouagadougou where he will take over the Neonatology Unit.

At the Neonatal Pathology of CMSC at admission and at discharge all patients were registered and from this database of infants we derived the following data used in the present analysis: date of birth, sex, gestational age, width, length, head circumference, hospital of birth, diagnosis, discharge date, type of discharge as alive, or dead. We compared the survival rates at the start of our project in 2005 with the data from the years 2008 to 2010 overall and then stratifying for gestational age, birth weight [very low birth weight (VLBW) <1,500 g, extremely low birth weight (ELBW) <1,000 g], and place of birth (at CMSC, the inborn; anywhere else, outborn).

## Results

As reported in Table 1, since 2005 we registered an increase in the total number of infants hospitalized, a steady increase in survival and a progressive increase of outborn infants. This is because the CMSC has become the reference center for neonatal care in the country.

**Table 1:** Major crude outcome measures for all hospitalized patients in the study period (2005, 2008–2010)

Calendar year	2005	2008	2009	2010	*p* value
Hospitalized patients					
*n*	887	1,217	1,278	1,472	–
Changes	Ref	1.37	1.44	1.66	
Survived patients					
*n*	500	674	787	902	0.004^a^
Rate	0.56	0.55	0.62	0.61	
95 % CI	0.53–0.60	0.53–0.58	0.59–0.64	0.59–0.64	
Outborn					
*n*	NA	961	1,097	1,211	0.043^a^
Rate	NA	0.79	0.86	0.82	

*Outborn* babies borned in other hospitals or facilities

^a^Trend test chi-square *p* value (1df)

Even when we restricted our analyses to the neonatal population of VLBW, we also observe a large and constant increased number of treated patients (>400 pt/year) with a significant increase in survival from 23 to 50 %. These samples were not selected since the birth weight and the gestational age did not change much in the study period. This result is obviously much less encouraging in ELBW infants who require mechanical ventilation, path still not viable in our hospital (Table 2).

**Table 2:** Major crude outcome measures restricted to VLBW hospitalized patients in the study period (2005, 2008–2010)

Calendar year	2005	2008	2009	2010	*p* value
*n*	307	513	412	415	0.0003^a^
% of all points	34.6 %	42.2 %	32.2 %	28.2 %	
Birth weight (g)					
Mean	1,251.2	1,208	1,288	1,231	ns
95 % CI	1,211.7–1,290.7	1,183.2–1,232.8	1,268.6–1,307.4	1,218.0–1,244.0	
Gestational age (weeks)					
Mean	29.5	30.6	31.9	31.6	ns
95 % CI	29.3–29.7	30.3–30.9	31.6–32.2	31.4–31.8	
Survived patients					
*n*	71	185	184	207	<0.0001^a^
Rate	0.23	0.36	0.45	0.50	
95 % CI	0.18–0.28	0.32–0.40	0.40–0.50	0.45–0.55	

^a^Trend test chi-square *p* value (1df)

ns = non-statitically significant

We also made an estimate of the cost of our intervention, considering hiring and training of personnel, increased consumption of drugs (antibiotics, diuretics, analeptics, analgesics) and oxygen, and cost due to increased consumption of electricity.

The improved level of care and the observed increase in survival came at a reasonable price. Hiring and training of the personnel was the major spending issue, while drugs and electricity were less expensive than expected. However, most families could not pay even the modest fee charged by the hospital and thus almost 80 % of the patients were treated at no cost for the parents.

To get more state funds for the Neonatal Pathology Unit, the Direction of the CMSC is trying to implement educational activities for students and staff of the other Birth Centers as well as using patronage of foreign organizations.

## Discussion

We were able to show that it is possible to increase survival of newborns, and especially premature newborns, in a developing country with low cost measures. In particular, survival of VLBW babies almost doubled from 23 % in 2005 to 50 % in 2010. This success was achieved mainly through training highly motivated staff in basic newborn care, including fluid management, positioning, monitored oxygen supply, and correct nutrition together with a dedicated patient chart system. Assigning each nurse her individual patients for whom she would be responsible during her shift, and assuring complete hand-over of all relevant information during shift change also contributed to strengthen responsibility and accountability of the operators. Improved hygiene and constant availability of supplemental oxygen were additional factors for this success. To motivate staff of the Pathology Neonatal Unit has been the main difficulty. A constant accompaniment of the nurses during our mission in the clinical examination and evaluation of the newborn, drafting procedures, educational programs, and preparation of medical records allows to improve the quality of neonatal assistance.

Further decrease in death rate can be achieved if mechanical ventilator could be available. Mechanical respiratory support remains a pivotal intervention for ELBW but without dedicated personnel trained in intubation and mechanical ventilation, and a guaranteed continuous supply in electricity, oxygen, and pressurized air, this much more expensive intervention was not possible. In this group of patients, delivery room procedures and a seamless transfer from the delivery room to the neonatal care unit are further critical steps which need improvement.

We now aim to implement emergency procedures with clear task assignment (who does what) to tackle this issue. The availability of additional drugs (like aminophylline and caffeine citrate) and low intensity ventilator support (like nasal CPAP) may improve the outcome for ELBW in the near future.

It is interesting to note, how the percentage of outborn patients increased over the years to over 80 %. It shows the excellent reputation of the CMSC, with birth clinics not only in the capital, but all over the country referring newborn babies for specialized care, making it the reference center for neonatal care in the country. On the other hand this also underlines the need to improve levels of care [8] in the other delivery clinics and obstetrics of the capital and the country. However, this also reflects the fact that the Camillian fathers provide care free of charge for all those in need, but decreased funding and increased cost make this ever more difficult for them.

For the time being, sustainability of this project for the coming year is provided by a generous donation from a private foundation. This will allow further visits that will aim not only to perpetuate achieved success and introduce further treatments to increase survival [9], but also to train staff of other birth clinics to increase mother and child health by increasing the number of well-performed monitoring visits for postpartum mothers and newborns [10]. In the last mission we started a training program involving the practitioners from other State Hospitals to improve the neonatal care levels.

## Conclusions

In extreme settings, even simple solutions can dramatically improve survival among premature newborns, the most at risk among those most at risk. Long lasting support is essential to reach durable improvements. Successful training of local staff [11] is not a one-time effort, but for trainers to be accepted and skills not only to be learned but to become routine and thus regularly adopted, repeated interventions, possibly by the same team are needed. Furthermore in an extremely resource-poor setting, every improvement, even the least expensive, has a price tag attached, and for parents that are truly unable to pay 1 EURO, someone else needs to cover the cost.

Thus external sources of funding and support from the local health system (providing for example skilled personnel and reimbursements for good health care) are important for the development of a functioning health system. It is necessary to specifically identify companies and hospitals that offer a partnership for a fixed period, with the possibility to verify the clinical progress and welfare ([12], dr Ouedraogo communication).

The Millennium Development Goals for maternal and child health (MDG 4 and 5) are to reduce by two-thirds, between 1990 and 2015. The child mortality under 5 years (U5MR: from 93 children of every 1,000 dying in 1990 to 31 of every 1,000 in 2015) is expected to reduce by three quarters, between 1990 and 2015, the maternal mortality rate (MMR). The indicators are: immunized against measles (%) within the first year (IMR), assisted deliveries (%) by qualified personnel, U5MR and MMR. According to the current trend of annual improvement, the fourth Millennium Development Goal (MDG 4) will be reached in 2045 rather than in 2015. Without an important reduction in the mortality during the first 7 days of life, we will not reach goal 4 of the “United Nations’ Millennium Development Goals” [13].

## References

[1] Lawn JE, Cousens S, Zupan J (2005). 4 million neonatal deaths: when? Where? Why?. Lancet.

[2] Opportunities for Africa’s newborns: Practical data, policy and programmatic support for newborn care in Africa. The Partnership for Maternal, Newborn and Child Health, 2006

[3] La condizione dell’infanzia nel mondo 2009, Unicef

[4] Millennium Development Goals Reports, ONU 2010

[5] Ibrahim SA, Babiker AG, Amin IK, Omer MI, Rushwan H (1994). Factors associated with high risk of perinatal and neonatal mortality: an interim report on a prospective community-based study in rural Sudan. Paediatr Perinat Epidemiol.

[6] McClure EM, Carlo WA, Wright LL, Chomba E, Lincetto O, Bann C (2007). Evaluation of the educational impact of the WHO Essential Newborn Care Course in Zambia. Acta Paediatr.

[7] Carlo WA, Wright LL, Chomba E (2009). Educational impact of the neonatal resuscitation program in low-risk delivery centers in a developing country. J Pediatr.

[8] Kwast BE (1996). Reduction of maternal and perinatal mortality in rural and peri-urban settings: what works?. Eur J Obstet Gynecol Reprod Biol.

[9] Darmstadt GL, Black RE, Santosham M (2000). Research priorities and postpartum care strategies for the prevention and optimal management of neonatal infections in less developed countries. Pediatr Infect Dis J.

[10] Carlo WA, Goudar SS, Jehan I (2010). Newborn-care training and perinatal mortality in developing countries. N Engl J Med.

[11] Uxa F, Bacci A, Mangiaterra V, Chiaffoni GP (2006). Essential newborn care training activities: 8 years of experience in Eastern European, Caucasian and Central Asian countries. Semin Fetal Neonatal Med.

[12] Atti del I° Workshop: “Cure neonatali nei paesi del terzo mondo: esperienze, confronti e strategie”, Parma 2010

[13] World Health Organization (2010). Neonatal and perinatal mortality: country, regional and global estimates 2006.

